# Tempol, a Superoxide Dismutase Mimetic Agent, Ameliorates Cisplatin-Induced Nephrotoxicity through Alleviation of Mitochondrial Dysfunction in Mice

**DOI:** 10.1371/journal.pone.0108889

**Published:** 2014-10-01

**Authors:** Lamiaa A. Ahmed, Nagwa I. Shehata, Noha F. Abdelkader, Mahmoud M. Khattab

**Affiliations:** 1 Department of Pharmacology and Toxicology, Faculty of Pharmacy, Cairo University, Cairo, Egypt; 2 Department of Biochemistry, Faculty of Pharmacy, Cairo University, Cairo, Egypt; University of Pecs Medical School, Hungary

## Abstract

**Background:**

Mitochondrial dysfunction is a crucial mechanism by which cisplatin, a potent chemotherapeutic agent, causes nephrotoxicity where mitochondrial electron transport complexes are shifted mostly toward imbalanced reactive oxygen species versus energy production. In the present study, the protective role of tempol, a membrane-permeable superoxide dismutase mimetic agent, was evaluated on mitochondrial dysfunction and the subsequent damage induced by cisplatin nephrotoxicity in mice.

**Methods and Findings:**

Nephrotoxicity was assessed 72 h after a single i.p. injection of cisplatin (25 mg/kg) with or without oral administration of tempol (100 mg/kg/day). Serum creatinine and urea as well as glucosuria and proteinuria were evaluated. Both kidneys were isolated for estimation of oxidative stress markers, adenosine triphosphate (ATP) content and caspase-3 activity. Moreover, mitochondrial oxidative phosphorylation capacity, complexes I–IV activities and mitochondrial nitric oxide synthase (mNOS) protein expression were measured along with histological examinations of renal tubular damage and mitochondrial ultrastructural changes. Tempol was effective against cisplatin-induced elevation of serum creatinine and urea as well as glucosuria and proteinuria. Moreover, pretreatment with tempol notably inhibited cisplatin-induced oxidative stress and disruption of mitochondrial function by restoring mitochondrial oxidative phosphorylation, complexes I and III activities, mNOS protein expression and ATP content. Tempol also provided significant protection against apoptosis, tubular damage and mitochondrial ultrastructural changes. Interestingly, tempol did not interfere with the cytotoxic effect of cisplatin against the growth of solid Ehrlich carcinoma.

**Conclusion:**

This study highlights the potential role of tempol in inhibiting cisplatin-induced nephrotoxicity without affecting its antitumor activity via amelioration of oxidative stress and mitochondrial dysfunction.

## Introduction

Cisplatin is an effective chemotherapeutic agent that is widely used against several types of solid tumors. However, its clinical use is limited by its potent nephrotoxicity [1], [2]. This nephrotoxicity seems to be related to accumulation of cisplatin more predominantly in the kidney than other tissues because it is the major route of its excretion [3].

Cisplatin nephrotoxicity has been recognized as a complex multifactorial process that includes oxidative stress, mitochondrial dysfunction and alteration of signal transduction pathways involved in apoptosis [4]. Increased oxidative stress is one of the earliest features associated with the development of cisplatin-induced nephropathy [5]. Several investigators have demonstrated that interaction of cisplatin with SH-groups leads to glutathione (GSH) depletion, along with a decline of cellular antioxidant system and accumulation of reactive oxygen species (ROS) or their products [6], [7].

Mitochondrial injury seems to play an important role in cisplatin-induced nephrotoxicity. Several functional and structural alterations of the mitochondria have been observed in cell cultures and in vivo animal models of cisplatin nephrotoxicity [8], [9]. This was evidenced by decreased mitochondrial mass with reduction of activities of oxidative phosphorylation complexes and manganese superoxide dismutase (MnSOD). This selectivity for mitochondria is probably caused by the accumulation of positively charged aquated complexes of cisplatin in the negatively charged inner space of the mitochondria [10]. Thus, increased oxidative stress in cisplatin nephrotoxicity may be simply a consequence of disrupted respiratory chain and decreased antioxidant activity since mitochondria are a major source and target for damage by ROS [11].

Oxidative stress and mitochondrial damage have been proposed as important factors that are involved in the activation of apoptotic pathway and cisplatin-induced cell death in vitro [12] as well as in vivo [13]. These events, together, result in the loss of renal function during cisplatin nephrotoxicity, triggering acute renal failure and tubular injury [4]. A wide variety of antioxidants have been reported to exhibit protective effects against the deleterious effects of cisplatin-induced nephrotoxicity [14]–[17]. As superoxide anions are the major injurious oxidant species generated by mitochondria, earlier studies have focused on the protective role for mitochondrial localized MnSOD in several models of free radicals-mediated cell injury [18]–[20].

Tempol (4-hydroxy tempo) is a membrane-permeable radical scavenger which has SOD and catalase activities. Tempol has been reported experimentally to ameliorate oxidative stress-mediated renal dysfunction and glomerular injury [21]. Moreover, tempol ameliorated endothelial cell dysfunction in diabetic rats [22] and reduced infarct size in an experimental model of regional myocardial ischemia/reperfusion [23]. A phase I clinical trial in patients receiving whole brain radiotherapy suggested that tempol may be effective at preventing radiation-induced alopecia with only mild (grade I and II) toxicity [24].

Tempol, as an antioxidant, has been previously demonstrated to prevent injury induced by cisplatin using established renal epithelial cell line, LLC-PK1 [25]. However, no study has investigated the effect of tempol in an in vivo experimental model of cisplatin-induced nephrotoxicity in addition to mitochondrial role in its possible mediated protection. Therefore, the goal of the present study was directed to examine the implication of this membrane-permeable SOD-mimetic agent, tempol, in the prevention of mitochondrial dysfunction in cisplatin-induced nephrotoxicity. Moreover, the effect of tempol on cisplatin antineoplastic efficacy was investigated.

## Materials and Methods

### 1. Animals

Male Swiss albino mice weighing 21–24 g were obtained from the animal facility of Faculty of Pharmacy, Cairo University, Egypt. Animals were housed under controlled environmental conditions at constant temperature (25±2°C) and a 12/12 h light/dark cycle. Mice were allowed standard chow diet and water ad libitum. The investigation complies with the *Guide for Care and Use of Laboratory Animals* published by the US National Institutes of Health (NIH Publication No. 85–23, revised 1996) and was approved by the Ethics Committee for Animal Experimentation at Faculty of Pharmacy, Cairo University (Permit Number: PT 983).

### 2. Chemicals

Tempol, cisplatin, fine chemicals and reagents, unless otherwise specified, were obtained from Sigma-Aldrich Chemical Co. (St. Louis, MO, USA).

### 3. Experimental Design

For selection of cisplatin dose, two doses have been examined; 12 mg/kg [26] and 25 mg/kg [27]. After 72 h, a single i.p. injection of cisplatin (25 mg/kg) caused pronounced nephrotoxicity and effectively suppressed the growth of solid Ehrlich carcinoma in mice (data not shown) and thus was selected to complete the present study. For the main study, fifty mice were randomly divided into four groups. Group I (n = 12) served as a normal group. Group II (n = 12) received tempol (100 mg/kg/day) orally for 4 successive days. The used dose of tempol was selected from previous studies [28], [29], [30] based on its safety and effectiveness as a protective agent in other models. Group III (n = 14) received a single i.p. injection of cisplatin (25 mg/kg). Group IV (n = 12) received tempol as in group II and cisplatin as in group III one day after tempol administration. Mice were weighed at the beginning of experiment and 72 h after receiving cisplatin. For measurement of glucosuria and proteinuria; a urine sample was obtained from each animal by massaging the lower abdomen of mice directed to the meatus [31]. Blood was then collected under anesthesia from the retro-orbital sinus using non heparinized capillary tubes for serum separation. At the end of the experiment, animals were euthanized and the whole kidneys were rapidly excised, washed with ice-cold saline, dried and weighed. For each group, two sets of experiments were conducted; one for biochemical investigations and the other (n = 4) for histological examinations.

### 4. Biochemical measurements

One kidney was used for mitochondrial separation while the other was homogenized in ice-cold saline to prepare 10% homogenate for determination of adenosine triphosphate (ATP) content as well as caspase-3 activity.

#### 4.1. Serum creatinine and urea levels

Serum creatinine and urea levels were estimated using commercial kits purchased from Stanbio Chemicals (USA) and Quimica Clinica Aplicada (Spain), respectively. Procedures were performed according to the manufacturer’s instructions. Results were expressed as mg/dl.

#### 4.2. Glucosuria and proteinuria

Urinary glucose and protein levels were estimated using commercial kits purchased from Biodiagnostic, Egypt. Procedures were performed according to the manufacturer’s instructions. Urinary creatinine was also evaluated as previously mentioned for estimation of urinary glucose/creatinine ratio and protein/creatinine ratio to compensate for variations in urine concentration in the used samples.

#### 4.3. Isolation of mitochondria

Mitochondrial isolation was performed as mentioned in a previous study [32]. In brief, kidney was homogenized in 0.7 M Tris-HCl buffer (pH 7.4) containing 0.25 M sucrose and centrifuged at 2500×g for 10 min at 4°C to remove nuclei and unbroken cells. The supernatant fluid was decanted into eppendorf tubes and centrifuged at 10,000×g for 10 min at 4°C to form primary mitochondrial pellet. The supernatant fluid (postmitochondrial fraction) was removed for further analysis and the pellet was gently resuspended in 0.5 ml Tris-sucrose buffer for washing. The pellet was recentrifuged and the supernatant fluid was decanted. This washing cycle was repeated three times to improve the degree of mitochondrial purity. The final mitochondrial pellet was resuspended in Tris-sucrose buffer. The fresh mitochondrial suspension was used for estimation of oxidative phosphorylation capacity. Mitochondrial and postmitochondrial fractions were used for assessment of the levels of lipid peroxidation products and GSH as well as the activities of antioxidant enzymes; SOD and catalase in addition to complexes I–IV activities in mitochondrial fraction. The protein contents of tissue homogenate, mitochondrial and postmitochondrial fractions were determined using the method of Lowry et al. [33].

#### 4.4. Lipid peroxidation products

Mitochondrial and postmitochondrial lipid peroxidation products were estimated by determination of the level of thiobarbituric acid reactive substances (TBARS) that was measured according to the assay of Buege and Aust [34] and expressed as nmol/mg protein.

#### 4.5. Reduced glutathione

Mitochondrial and postmitochondrial GSH contents were determined using the method of Beutler et al. [35] and results were expressed as nmol/100 mg protein.

#### 4.6. Superoxide dismutase activity

Mitochondrial and postmitochondrial SOD activities were assessed according to the method of Marklund [36]. Results were expressed as U/mg protein. One unit is defined as the amount of enzyme that produces 50% inhibition of pyrogallol autooxidation.

#### 4.7. Catalase activity

Mitochondrial and postmitochondrial catalase activities were assessed according to the method of Abei [37]. Results were expressed as U/mg protein. One unit of catalase activity is defined as the amount of enzyme that degrades 1 µmol hydrogen peroxide (H_2_O_2_) per min at 25°C.

#### 4.8. Mitochondrial oxidative phosphorylation capacity

Mitochondrial phosphate utilization was assayed following the method of Hinkle [38] and Banerjee et al. [39] which depends on inorganic phosphate (Pi) utilization by fresh mitochondrial suspension after incubation in a suitable reaction buffer. The amount of Pi in the supernatant was estimated according to the method of Bagh et al. [40] and results were expressed as nmol Pi/min/mg protein.

#### 4.9. Estimation of mitochondrial nitric oxide synthase (mNOS) protein expression

A part of mitochondrial pellet was resuspended in lysis buffer and quantified for protein levels using a commercial assay kit (Thermo Fisher Scientific Inc., USA). An aliquot of 20 µg protein from each sample was separated on 8% sodium dodecyl sulphate-polyacrylamide gel electrophoresis and transferred to a nitrocellulose membrane (Amersham Bioscience, Piscataway, NJ, USA) using a semidry transfer apparatus (Bio-Rad, Hercules, CA, USA). Membranes were blocked with 5% nonfat milk in Tris- buffered saline with 0.05% Tween-20 (TBST) at 4°C overnight. The membranes were then washed with TBST and incubated with a 1∶2,000 dilution of anti-iNOS antibodies (Stressgen Biotechnologies, Victoria, British Columbia, Canada) for 1 h at room temperature with constant shaking. The filters were washed and subsequently probed with horseradish peroxidase-conjugated goat anti-mouse immunoglobulin (Amersham. Life Science Inc., USA). Chemiluminescence detection was performed with the Amersham detection kit according to the manufacturer’s protocols and exposed to X-ray film. The amount of NOS protein was quantified by densitometric analysis of the autoradiograms using a scanning laser densitometer (Biomed Instrument Inc., USA). Results were expressed as arbitrary units after normalization for beta-actin (β-actin) protein expression.

#### 4.10. Adenosine triphosphate content

ATP content was estimated according to the method of Lowry et al. [41] which depends on the increase in fluorescence due to the formed NADPH by reaction of ATP with glucose in the presence of NADP^+^ and glucose-6-phosphate dehydrogenase. Results were expressed as µmol/g wet tissue.

#### 4.11. Complexes I–IV activities

Complexes I–III activities were estimated according to the method of Sharman and Bondy [42]. For complex I (NADH-ubiquinone oxidoreductase) activity, the method involves the reduction of decylubiquinone to the corresponding quinol with the concurrent oxidation of NADH to NAD^+^ and the results were expressed as nmol NADH oxidized/min/mg protein. Complex II (succinate dehydrogenase) was assayed using phenazine methosulfate as an artificial electron acceptor from succinate and recording the increase in absorbance of ferrocytochrome-c where complex II activity was calculated as nmol succinate oxidized/min/mg protein. For estimating the activity of complex III (ubiquinol-cytochrome c oxidoreductase), the method depends on the oxidation of decylubiquinol to the corresponding quinone with the concurrent reduction of ferricytochrome-c to ferrocytochrome-c and its activity was computed as the initial reaction rate per mg protein. Finally, the activity of complex IV (cytochrome-c oxidase) was assayed using the method of Storrie and Madden [43]. This method is based on monitoring the decrease in absorbance of ferrocytochrome-c caused by its oxidation to ferricytochrome-c where its activity was calculated in nmol cytochrome-c oxidized/min/mg protein.

#### 4.12. Caspase-3 activity

Caspase-3 activity was estimated using caspase-3 colorimetric assay kit (R&D Systems. Inc, USA). Results were expressed as nmol p-nitroanilide (pNA)/h/mg protein.

### 5. Histological examinations

#### 5.1. Assessment of renal tubular damage

For light microscopic examination, the right kidney was separated, rinsed in ice-cold saline and immediately fixed in 10% formalin for 24 h. Specimens were processed for paraffin embedding and 5 µm sections were prepared. Sections were stained with haematoxylin and eosin (H&E) and examined microscopically (magnification x200). Images were captured and processed using Adobe Photoshop (version 8.0). Images were analyzed by two investigators. Histological changes were evaluated semiquantitatively by a pathologist unaware of the type of treatment. For quantification of tubular injury score, sections were assessed by counting the percentage of tubular necrosis in the outer stripe of outer medulla (OSOM) using the following scoring criteria: 0 = normal; 1 = <10%; 2 = 10–25%; 3 = 26–75%; 4 = >75%. Tubular necrosis was defined as the loss of the proximal tubular brush border, blebbing of apical membranes, tubular epithelial cell detachment from the basement membrane or intraluminal aggregation of cells and proteins [44]. A total of ten fields were scored from each sample and averaged. Scores from different sections were then summed up to obtain an average score per field for each group.

#### 5.2. Electron microscopic examination

Small pieces were separated from the left kidney, rinsed in ice-cold saline and cut into fragments (diameter = 1 mm). Fragments were then processed and ultra-thin sections were stained with uranyl acetate and lead citrate and examined with a transmission electron microscope (H-300, HITACHI, Japan) and photographed. Electron micrographs were taken systematically at ×2,000 and ×10,000 magnifications. Images were analyzed by two investigators. Percentage of mitochondrial cross-sectional area/cytoplasmic area was estimated where non-cytoplasmic area was first manually excluded from analysis. The optical density threshold was manually set to include all mitochondria and then the percentage area of mitochondria was calculated per cytoplasmic volume [9]. Moreover, damage of each mitochondrion was scored on a 0–3 scale according to the extent of morphological alterations [45]; 0 = normal, 1 = marked swelling (separation of cristae, decreased matrix density), 2 = massive swelling with architectural disruption and 3 = findings as grade 2 plus rupture of mitochondrial membranes. Average values from two observers were calculated and approximately 150 mitochondria/sample were graded.

### 6. Effect of cisplatin, tempol and their combination on the growth of solid Ehrlich carcinoma in mice

In this experiment, 2×10^6^ Ehrlich ascites carcinoma cells were transplanted subcutaneously in the right thigh of the lower limb of each mouse. Mice with a palpable solid tumor mass (100 mm^3^) that developed within 7 days after implantation were divided into four groups, 6 animals each. Two groups were injected with either cisplatin as previously mentioned in the experimental design or an equivalent volume of normal saline. The other two groups received tempol orally in a dose of 100 mg/kg/day or a combination of tempol and cisplatin. The change in tumor volume was measured after 3 days using a Vernier caliper and was calculated by the following formula according to Osman et al. [46]:
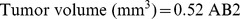
where A is the minor tumor axis and B is the major axis.

### 7. Statistical analysis

All data obtained were presented as mean ± S.E.M. Results were analyzed using one way analysis of variance test (One-way ANOVA) followed by Student-Newman-Keuls multiple comparison test. Statistical analysis was performed using GraphPad Prism software (version 5). For all the statistical tests, the level of significance was fixed at p<0.05.

## Results

### 1. Effect of tempol on cisplatin-induced changes in mortality, body weight and relative kidney weight

Cisplatin-treated group showed significant decrease in survival% (71%) and body weight (20.42±0.98 vs. 23.33±0.70 g) as well as significant increase in the relative kidney weight (1.64±0.03 vs. 1.10±0.02); which were pronouncedly alleviated with tempol pretreatment (Table 1).

**Table 1:** Effect of tempol on cisplatin-induced changes in mortality, initial body weight (IBW), final body weight (FBW), relative kidney weight, serum creatinine and urea, glucosuria and proteinuria in mice.

Groups	Mortality%	IBW (g)	FBW (g)	Relativekidneyweight (%)	Serumcreatinine(mg/dl)	Serumurea(mg/dl)	Urinaryglucose/creatinineratio	Urinaryprotein/creatinineratio
**Normal**	0%	22.17±0.61	24.64±1.13	1.10±0.02	0.567±0.083	33.22±2.09	0.34±0.07	1.99±0.15
**Tempol**	0%	22.33±0.76	27.83±1.37	1.06±0.04	0.442±0.046	26.55±1.46	0.38±0.07	1.67±0.10
**Cisplatin**	29%	23.33±0.70	20.42±0.98*	1.64±0.03*	2.786±0.367*	310.30±33.19*	13.14±1.93*	7.97±0.99*
**Cisplatin + Tempol**	0%	22.17±0.65	23.14±1.12	1.14±0.03^#^	0.633±0.089^#^	41.60±3.91^#^	0.67±0.03^#^	2.37±0.21^#^

Relative kidney weight was calculated as the percentage of weights of kidneys divided by the final body weight of the mice. Mice were studied 72 h after a single i.p. injection of cisplatin (25 mg/kg). Tempol (100 mg/kg/day) was given orally for 4 days starting one day before cisplatin injection. Glucosuria and proteinuria were evaluated as urinary glucose/creatinine ratio and urinary protein/creatinine ratio. Each value represents the mean of 10–12 mice ± S.E.M. *p<0.05 vs. normal, ^#^ *p<0.05 vs*. cisplatin.

### 2. Effect of tempol on cisplatin-induced renal dysfunction

Cisplatin raised both serum creatinine and urea levels by five and nine folds, respectively compared to the normal group. Cisplatin-treated animals also exhibited a significant renal tubular dysfunction as evidenced by marked glucosuria and proteinuria (Table 1). Pretreatment with tempol attenuated cisplatin-induced renal dysfunction as demonstrated by normalization of serum creatinine level and significant reduction of serum urea, glucosuria and proteinuria compared to cisplatin group (Table 1).

### 3. Effect of tempol on cisplatin-induced renal alterations in oxidative stress biomarkers in both mitochondrial and postmitochondrial fractions

**Figure 1:**
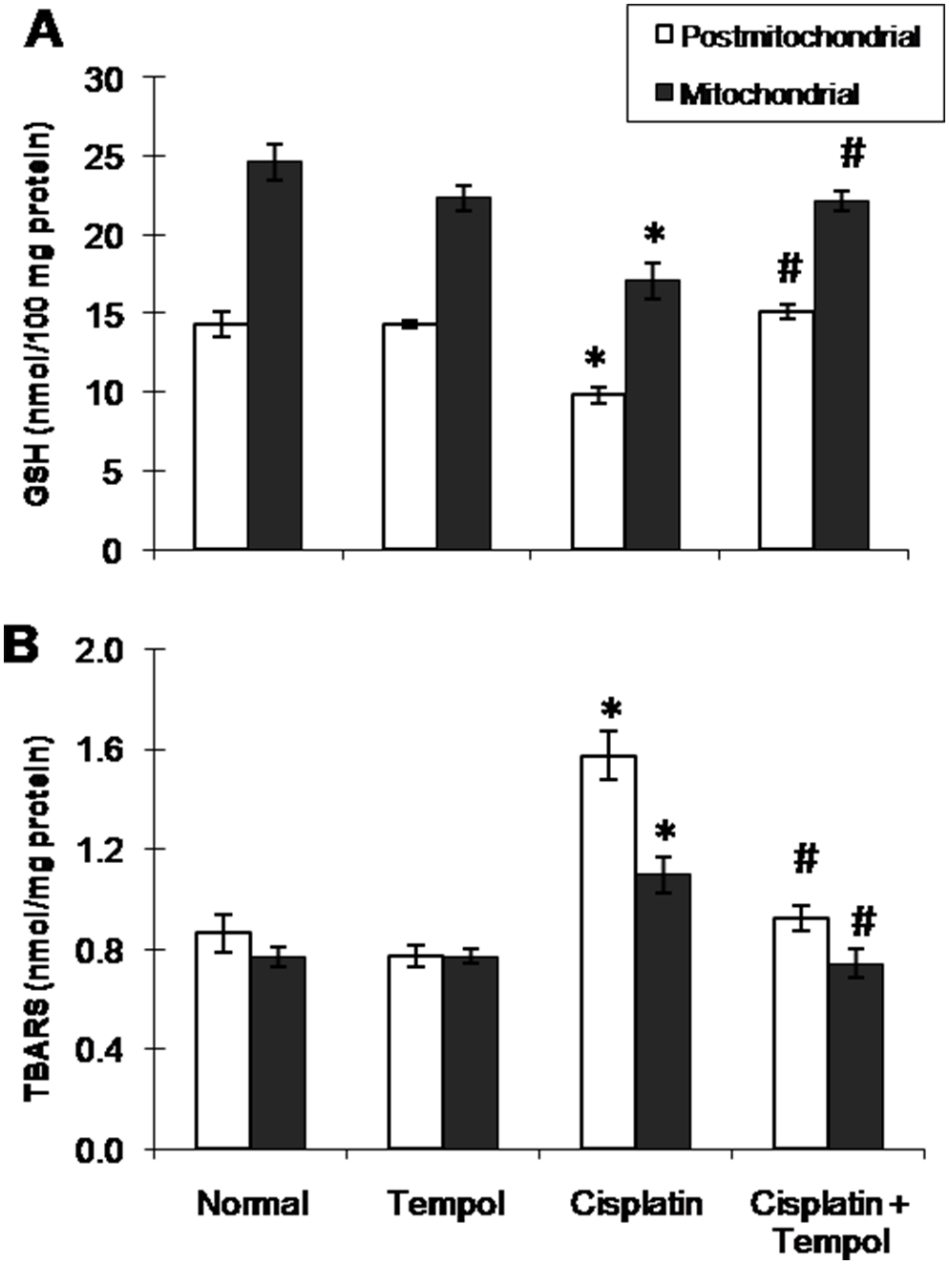
Effect of tempol on cisplatin-induced changes in oxidative stress markers in postmitochondrial and mitochondrial fractions in renal tissues of mice. (A) Reduced glutathione (GSH). (B) Thiobarbituric acid reactive substances (TBARS). Mice were studied 72 h after a single i.p. injection of cisplatin (25 mg/kg). Tempol (100 mg/kg/day) was given orally for 4 days starting one day before cisplatin injection. Each value represents the mean of 6–8 mice ± S.E.M. *p<0.05 vs. normal, ^#^ *p<0.05 vs*. cisplatin.

**Figure 2:**
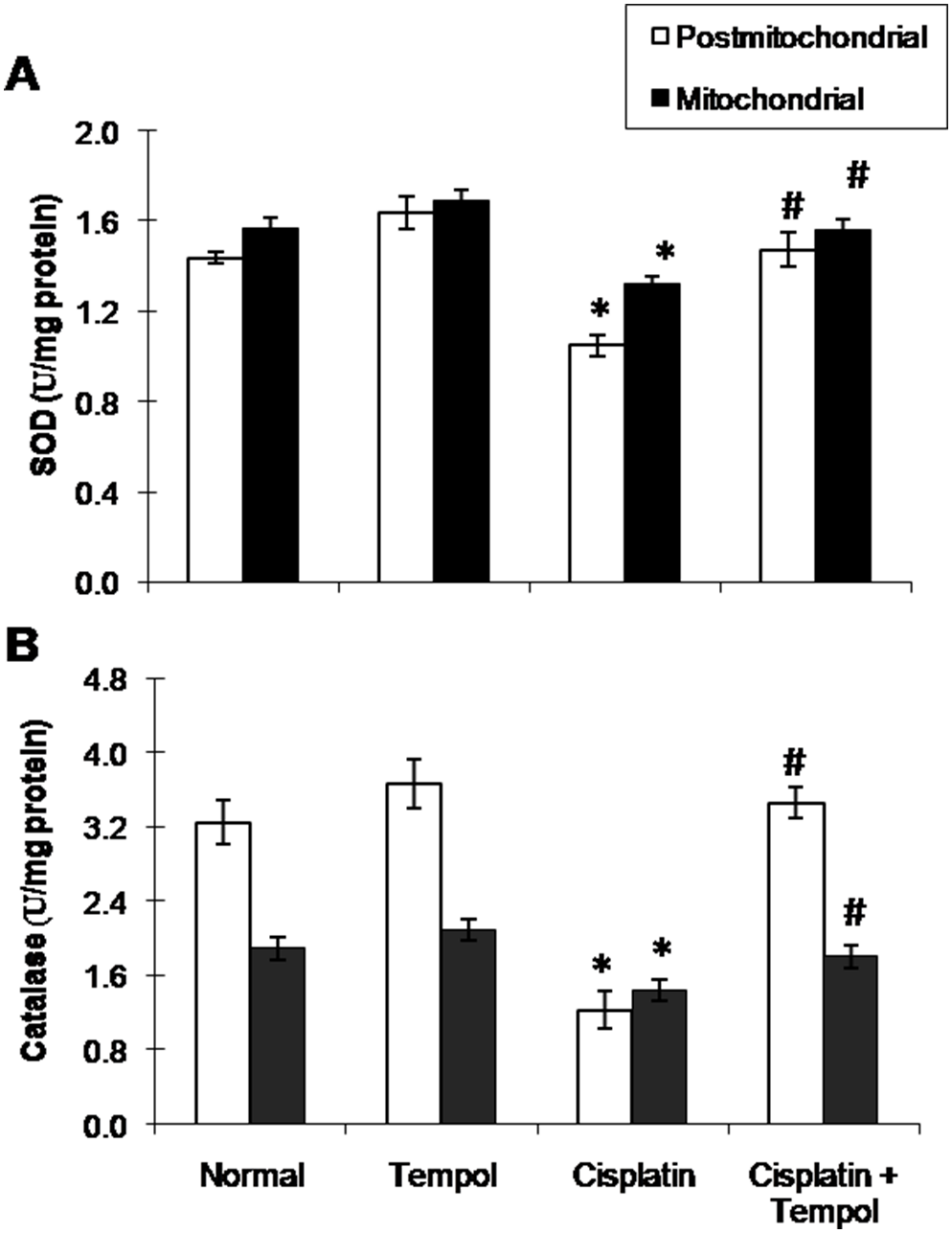
Effect of tempol on cisplatin-induced changes in antioxidant enzymes activities in postmitochondrial and mitochondrial fractions in renal tissues of mice. (A) Superoxide dismutase (SOD). (B) Catalase. Mice were studied 72 h after a single i.p. injection of cisplatin (25 mg/kg). Tempol (100 mg/kg/day) was given orally for 4 days starting one day before cisplatin injection. Each value represents the mean of 6–8 mice ± S.E.M. *p<0.05 vs. normal, ^#^ *p<0.05 vs*. cisplatin.

Cisplatin-treated group showed significant elevation of TBARS content and significant reduction of GSH content (Fig. 1) as well as SOD and catalase activities (Fig. 2) in both postmitochondrial and mitochondrial fractions. Tempol pretreatment alleviated the previously mentioned changes induced by the state of oxidative stress.

### 4. Effect of tempol on cisplatin-induced alterations in renal oxidative phosphorylation, complexes I-IV activities and ATP production

The enhanced state of oxidative stress in cisplatin-treated group was associated with significant reduction of mitochondrial oxidative phosphorylation capacity and complexes I and III activities. These changes were correlated with a pronounced reduction of ATP content (Table 2). On the other hand, tempol significantly alleviated the aforementioned disruption of mitochondrial function. Interestingly, tempol administration to normal mice significantly increased mitochondrial complex I activity compared to the normal value.

**Table 2:** Effect of tempol on cisplatin-induced changes in mitochondrial complexes I–IVactivities, oxidative phosphorylation capacity and adenosine triphosphate (ATP) content in renal tissues of mice.

Groups	Complex I(nmol/min/mg protein)	Complex II(nmol/min/mg protein)	Complex III(nmol/min/mg protein)	Complex IV(nmol/min/mg protein)	Oxidativephosphorylationcapacity (nmol Pi/min/mgprotein)	ATP(µmol/gwet tissue)
**Normal**	15.87±1.23	67.93±5.07	1332.00±110.2	202.50±13.42	695.00±61.90	13.19±0.84
**Tempol**	23.79±2.05*	76.57±8.55	1118.00±99.75	207.10±8.58	715.00±52.14	10.80±0.74
**Cisplatin**	6.42±0.56*	60.65±6.48	889.70±89.99*	167.20±6.19	230.00±21.60*	6.28±0.56*
**Cisplatin +** **Tempol**	12.61±1.15^#^	68.16±6.20	1337.00±95.10^#^	191.40±10.66	586.00±49.86^#^	11.53±0.52^#^

Mice were studied 72 h after a single i.p. injection of cisplatin (25 mg/kg). Tempol (100 mg/kg/day) was given orally for 4 days starting one day before cisplatin injection. Each value represents the mean of 6–8 mice ± S.E.M. *p<0.05 vs. normal, ^#^ *p<0.05 vs*. cisplatin.

### 5. Effect of tempol on cisplatin-induced elevation of renal caspase-3 activity and mNOS protein expression

**Figure 3:**
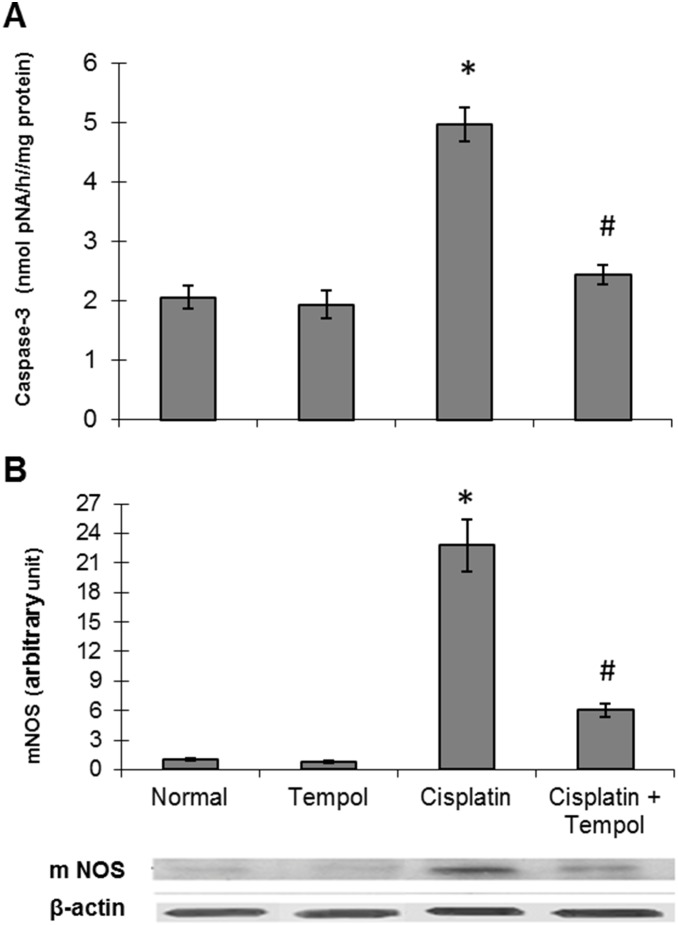
Effect of tempol on cisplatin-induced changes in caspase-3 activity and mitochondrial nitric oxide synthase (mNOS) protein expression in renal tissues of mice. (A) Caspase-3 activity. (B) mNOS protein expression. Mice were studied 72 h after a single i.p. injection of cisplatin (25 mg/kg). Tempol (100 mg/kg/day) was given orally for 4 days starting one day before cisplatin injection. Each value represents the mean of 6–8 mice ± S.E.M. *p<0.05 vs. normal, ^#^ *p<0.05 vs*. cisplatin.

Cisplatin-induced mitochondrial disruption was accompanied by significant elevation of caspase-3 activity and mNOS protein expression which was attenuated by tempol pretreatment (Fig. 3).

### 6. Effect of tempol on renal tubular damage and mitochondrial overall injury

**Figure 4:**
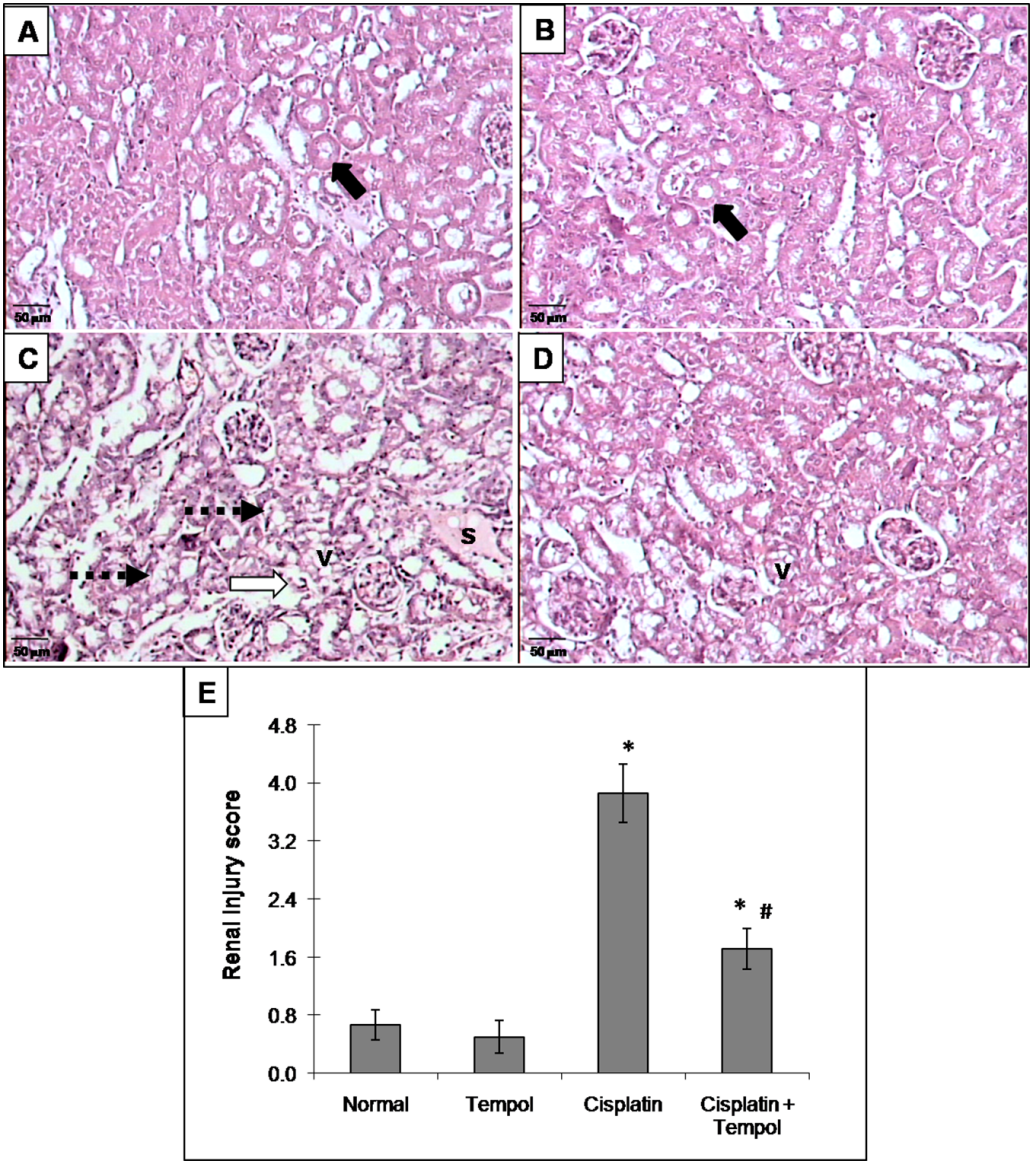
Effect of tempol on cisplatin-induced changes in light microscopic examination (H&E x200) in renal tissues of mice. (A) normal group and (B) tempol-treated group show normal non-affected tubular epithelium (thick dark arrow). (C) cisplatin-treated group shows severe tubular damage as revealed by acute tubular necrosis (dashed thick arrow), wide tubular epithelial vacuolation (v), apoptotic tubular epithelium (thick white arrow) and cast formation (s). (D) tempol and cisplatin-treated group shows more or less normal renal tubules with minimal focal vacuolation of the tubular epithelium (v). (E) renal injury score. Mice were studied 72 h after a single i.p. injection of cisplatin (25 mg/kg). Tempol (100 mg/kg/day) was given orally for 4 days starting one day before cisplatin injection. Each renal injury score value represents the mean of 4 mice ± S.E.M. *p<0.05 vs. normal, ^#^ *p<0.05 vs*. cisplatin.

**Figure 5:**
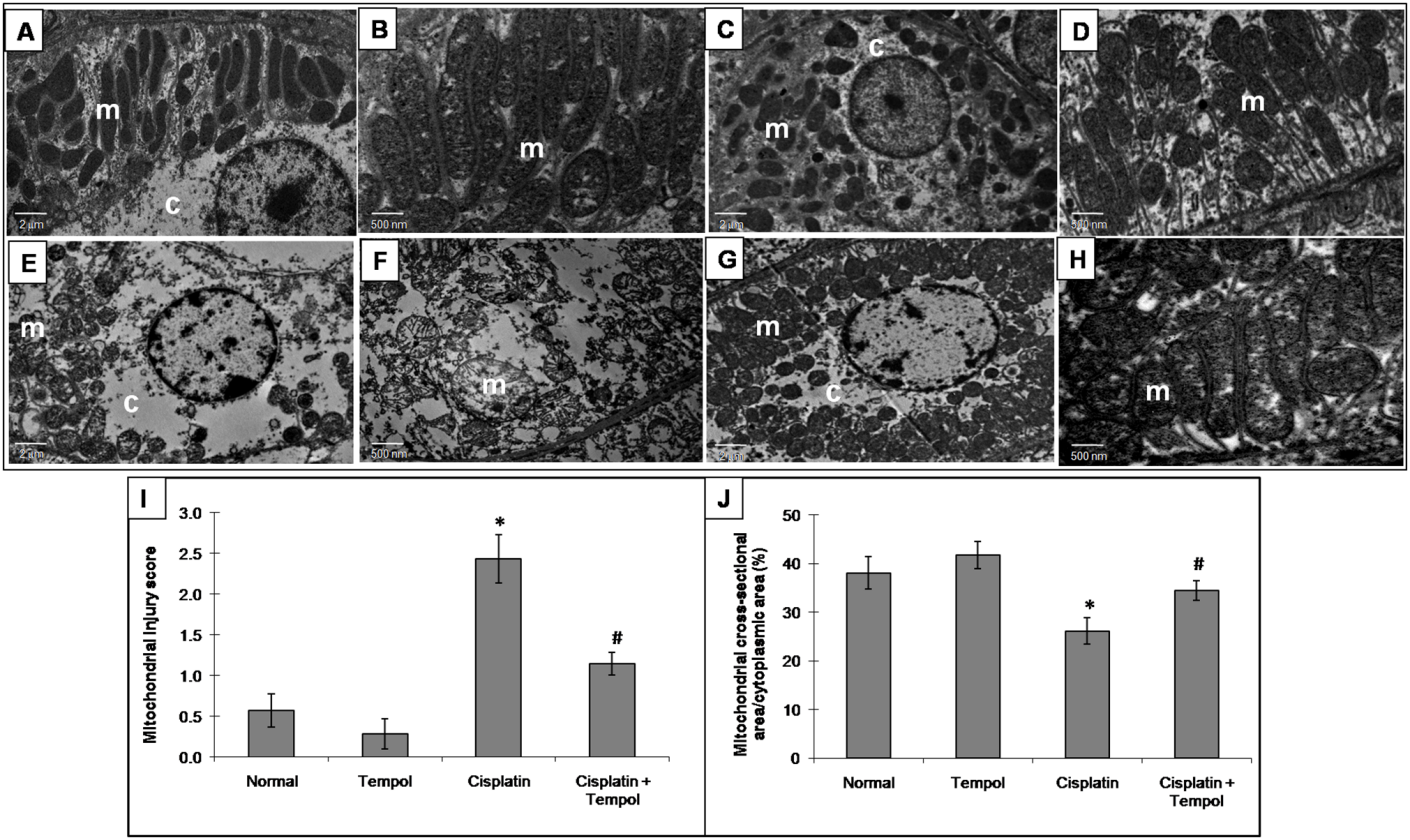
Effect of tempol on cisplatin-induced changes in mitochondrial ultrastructural examination of renal tissues in mice. Photomicrographs are representative specimens which show mitochondria (m) and cytoplasm (c) from normal group (A x2,000 magnification; B x10,000 magnification), tempol-treated group (C x2,000 magnification; D x10,000 magnification), cisplatin-treated group (E x2,000 magnification; F x10,000 magnification), tempol and cisplatin-treated group (G x2,000 magnification; H x10,000 magnification), mitochondrial overall injury score (I) and percentage of mitochondrial cross-sectional area/cytoplasmic area (J). Mice were studied 72 h after a single i.p. injection of cisplatin (25 mg/kg). Tempol (100 mg/kg/day) was given orally for 4 days starting one day before cisplatin injection. Each value represents the mean of 4 mice ± S.E.M. *p<0.05 vs. normal, ^#^ *p<0.05 vs*. cisplatin.

Cisplatin-treated group showed a marked tubular damage compared to the normal group. Pretreatment with tempol significantly decreased tubular damage score (Fig. 4). the mitochondrial overall injury score showed a marked increase in cisplatin-treated group represented by a decrease in the number of normal mitochondria with a concomitant increase in the proportion of mitochondria showing ultrastructural alterations. Moreover, cisplatin caused a significant decrease in the percentage of mitochondrial cross-sectional area/cytoplasmic area. Pretreatment with tempol preserved mitochondrial ultrastructural changes as revealed by a significant decrease in mitochondrial overall injury score together with a significant increase in the percentage of mitochondrial cross-sectional area/cytoplasmic area (Fig. 5).

### 7. Effect of cisplatin, tempol and their combination on the growth of solid Ehrlich carcinoma in mice

Treatment of mice with cisplatin (25 mg/kg) revealed a cessation of solid Ehrlich carcinoma tumor growth where the tumor volume was significantly decreased compared to the control group. Co-administration of tempol with cisplatin suppressed tumor growth in a similar pattern to cisplatin-treated animals. Therefore, tempol did not interfere with the cytotoxic effect of cisplatin against the growth of solid Ehrlich carcinoma (Table 3).

**Table 3:** Effect of tempol, cisplatin and their combination on the growth of solid Ehrlich carcinoma 72 h after starting treatment.

Groups	Ehrlich tumor (mm^3^)
**Control**	710.40±69.06
**Tempol**	665.43±64.98
**Cisplatin**	278.65±26.48^$^
**Cisplatin + Tempol**	248.59±23.96^$^

Mice with a palpable solid tumor mass (100 mm^3^) were given a single i.p. injection of cisplatin (25 mg/kg) or tempol orally in a dose of 100 mg/kg/day or a combination of tempol and cisplatin. The change in tumor volume was measured after 3 days using a Vernier caliper. Each value represents the mean of 5–6 mice ± S.E.M. ^$^ *p<0.05 vs*. control.

## Discussion

Despite being widely used, the use of cisplatin as chemotherapy is limited by its nephrotoxicity with about 25–35% of patients experiencing a significant decline of renal function after a single dose of cisplatin treatment [47], [48]. The goal of the present study was directed to examine the potential role of a membrane-permeable SOD-mimetic agent, tempol, in alleviating mitochondrial dysfunction in cisplatin-induced nephrotoxicity and to evaluate the effect of tempol on cisplatin antineoplastic efficacy. In the present investigation, mice treated with cisplatin showed significant decrease in body weight and % survival. Cisplatin-induced weight loss might be due to gastrointestinal toxicity and reduction of food ingestion [49]. Pretreatment with tempol pronouncedly alleviated the decrease in % survival and body weight in accordance with the amelioration of cisplatin nephrotoxicity as demonstrated in the present study.

The present data show that administration of cisplatin to mice caused marked elevation of serum creatinine and urea levels as well as significant glucosuria and proteinuria. Cisplatin causes acute renal failure due to its preferential accumulation within the proximal tubular cells in the outer medulla of the kidney [50], [51]. The alterations in glomerular function in cisplatin-treated mice may be secondary to the oxidative stress status observed in the present study, which induces mesangial cells contraction, alteration of filtration surface area and modification of ultrafiltration coefficient factors that decrease the glomerular filtration rate [52]. Pretreatment with tempol normalized serum creatinine level and caused significant reduction of serum urea, glucosuria and proteinuria compared to cisplatin group, indicating improvement of glomerular and tubular functions.

The present results clearly indicate a significant degree of oxidative stress in both mitochondrial and postmitochondrial fractions in renal tissues of cisplatin-treated mice. This was demonstrated by a significant elevation of TBARS content and a significant reduction of GSH content along with inhibition of SOD and catalase activities in both mitochondrial and postmitochondrial fractions. The enhanced state of oxidative stress in cisplatin-treated group was associated with significant reduction of mitochondrial oxidative phosphorylation capacity and complexes I and III activities together with significant elevation of mNOS protein expression and caspase-3 activity. This was correlated with a pronounced reduction of ATP content indicating marked deterioration of mitochondrial respiration and energy production. GSH is one of the essential components for maintaining cell integrity because of its reducing properties and participation in the cell metabolism. The depletion of the renal GSH level has been observed in response to oxidative stress caused by cisplatin treatment [53]. Platinum was shown to preferentially bind to GSH and protein thiol in kidneys after cisplatin treatment. The concentration of platinum-bound proteins was higher in the mitochondrial fraction than in the cytosolic fraction [54]. Formation of these complexes limits the amount of drug available for DNA binding and therefore, a positive correlation has been reported between GSH levels and resistance to cisplatin [55].

Cisplatin affects many enzymes that protect the cells from oxidative damage, among which Cu, Zn-SOD, Mn-SOD and catalase. SOD plays an important role in the dismutation of superoxide anions. Decreased SOD activity as observed in this study could lead to incomplete scavenging of superoxide anions that are produced during the normal metabolic process with further initiation and propagation of lipid peroxidation [56]. On the other side, the observed decrease in catalase activity after cisplatin administration could account for the disability of kidney to eliminate and scavenge toxic H_2_O_2_ and lipid peroxides. The inhibition of mitochondrial and postmitochondrial antioxidant enzyme activities may occur due to direct binding of cisplatin to essential sulfhydryl groups at the active sites of these enzymes and depletion of copper and manganese which are essential for SOD activity [57]. Meanwhile, the oxidative stress demonstrated in the current study may target multiple molecules in the cells and damage cell structural components such as lipids, proteins and other organelles where mitochondria are among the most affected ones.

Cisplatin exposure induces a mitochondria-dependent ROS generation that significantly contributes to cisplatin-induced nephrotoxicity [58]. Mitochondrial dysfunction has been reported to occur in rats following the depletion of cytosolic GSH with subsequent increase in lipid peroxidation in cisplatin-treated renal cortical slices [59]. Disturbances of the respiratory electron flow or the antioxidant defense mechanisms, can lead to an overproduction of superoxide anions in the respiratory chain of mitochondria by reaction of oxygen with iron-sulfur centers in complex I and by partially reduced ubiquinone and cytochrome b in complex III [60]. A previous study using cultured mouse proximal tubular cells demonstrated cisplatin-induced mitochondrial injury, as revealed by a decrease in mitochondrial succinate dehydrogenase activity, an induction of cytochrome c release, mitochondrial fragmentation and a reduction of complex IV protein [61]. In the present study, cisplatin-induced mitochondrial dysfunction was demonstrated by inhibition of complexes I and III of the respiratory chain. This result is in agreement with previous studies describing the inhibition of the mitochondrial respiratory chain complexes in vitro and the decline in mitochondrial respiratory activity in vivo by cisplatin [60], [62]. The inhibition of complexes I and III activities demonstrated in the current study was accompanied by deterioration of mitochondrial oxidative phosphorylation capacity, resulting in disruption of renal cellular energy production. The decrease in oxidative phosphorylation might be explained by the observed induction of mNOS in the present study which would increase NO production. Increased mitochondrial NO production possibly inhibits cytochrome c oxidase activity by competing with oxygen and also inhibits the electron transport chain at complexes I and III favoring superoxide formation. This in turn would increase peroxynitrite level, alter energy production by increased oxidation of mitochondrial proteins and induce macromolecular damage and cell death [63].

Partial ATP depletion as observed in the present study may constitute a common biochemical pathway that initiates a cascade of events leading to further cellular dysfunctions and activation of programmed cell death. This in turn may accelerate ROS formation by the damaged cells, which may contribute to an amplification of ROS-mediated cell death of the same cell or even the neighboring cells [60]. These biochemical alterations are consistent with renal tubular damage and mitochondrial ultrastructural changes evidenced in the present work by significant increase in tubular damage and mitochondrial overall injury scores with a concomitant decrease in the percentage of mitochondrial cross-sectional area/cytoplasmic area. Several studies have demonstrated a positive correlation between oxidative stress and apoptosis in experimentally-induced nephrotoxicity [4], [64], [65]. The elevation of caspase-3 activity, in the present study, reveals apoptosis and mitochondrial damage. Cisplatin-induced apoptosis in renal epithelial cells has been previously elucidated and is generally considered to be associated with caspase-3 activation which is the principal executioner caspase in renal tubular apoptosis [12].

Tempol pretreatment succeeded to alleviate the changes induced by the state of oxidative stress in both mitochondrial and postmitochondrial fractions in addition to amelioration of disruption of mitochondrial function and caspase-3 elevation. Several studies have previously demonstrated the role of antioxidants in prevention of cisplatin-induced nephrotoxicity [64], [66], [67]. Tempol has catalase and superoxide dismutase activities which can catalyze the removal of superoxide anions, limit hydroxyl radical formation from H_2_O_2_ and accept an electron to form the antioxidant hydroxylamine [68]. The antioxidant activities of tempol have been previously demonstrated experimentally in gentamicin and vancomycin-induced nephrotoxicity [69], [70]. Moreover, tempol has been previously shown to reduce renal cell damage caused by paraquat through its ROS scavenging activities [71]. Several molecular mechanisms for the antioxidant activity of tempol and other nitroxide derivatives have been proposed by inhibition of the iron-driven Fenton reaction with H_2_O_2_ by oxidizing transition metal ions, such as iron. Moreover, a direct superoxide anion scavenging activity of nitroxides has been reported [72].

Tempol, a membrane-permeable antioxidant, through its superoxide anion scavenging activity could prevent the increase in mitochondrial superoxide and H_2_O_2_ production and preserve the mitochondrial antioxidant enzyme activities including MnSOD and catalase. This was correlated with preservation of mitochondrial respiratory function (complexes I and III activities, oxidative phosphorylation capacity, mNOS and ATP production) and prevention of mitochondria-induced apoptosis as indicated by normalization of caspase-3 activity and histological examinations of renal tubules and mitochondria. Tempol treatment has previously restored mitochondrial membrane potential and reduced tissue oxidative damage in Atm-deficient mice, both in vitro and in vivo [73]. In addition, tempol protected rat proximal tubular cells against H_2_O_2_-induced cellular injury and death in a previous in vitro study [74].

In conclusion, the present study highlights the potential role of tempol in alleviating cisplatin-induced mitochondrial dysfunction without affecting its antitumor activity via reducing ROS formation, protecting electron transport complexes, favoring energy production and inhibiting apoptosis in addition to improving histological changes. The beneficial effect of tempol in cisplatin-induced nephrotoxicity is an important outcome which needs to be evaluated in clinical studies.
