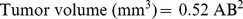# Correction: Tempol, a Superoxide Dismutase Mimetic Agent, Ameliorates Cisplatin-Induced Nephrotoxicity through Alleviation of Mitochondrial Dysfunction in Mice

**DOI:** 10.1371/journal.pone.0115983

**Published:** 2014-12-15

**Authors:** The PLOS ONE Staff

There is an error in the equation located in the “Effect of cisplatin, tempol and their combination on the growth of solid Ehrlich carcinoma in mice” section of the Materials and Methods. The 2 should be an exponent. This error was introduced while preparing the manuscript for publication. The publisher apologizes for this error. Please see the correct equation here.